# Vascular Inflammation and Cardiovascular Burden in Metastatic Breast Cancer Female Patients Receiving Hormonal Treatment and CDK 4/6 Inhibitors or Everolimus

**DOI:** 10.3389/fcvm.2021.638895

**Published:** 2021-02-23

**Authors:** Christos Papageorgiou, Flora Zagouri, Konstantinos Tampakis, Rebecca Georgakopoulou, Efstathios Manios, Pavlos Kafouris, Georgios Benetos, Iosif Koutagiar, Constantinos Anagnostopoulos, Meletios A. Dimopoulos, Konstantinos Toutouzas

**Affiliations:** ^1^Department of Clinical Therapeutics, Alexandra Hospital, School of Medicine, National and Kapodistrian University of Athens, Athens, Greece; ^2^Experimental Surgery and Translational Research, Biomedical Research Foundation Academy of Athens, Athens, Greece; ^3^Department of Informatics and Telecommunications, National and Kapodistrian University of Athens, Athens, Greece; ^4^1st Department of Cardiology, Hippokration Hospital, School of Medicine, National and Kapodistrian University of Athens, Athens, Greece

**Keywords:** vascular inflammation, remodeling and dysfunction, breast cancer, blood pressure, CDK 4/6 inhibitor, cardiovascular toxicity from anticancer drugs

## Abstract

**Background:** Chemotherapy regimens for breast cancer treatment can promote vascular dysfunction and lead to high cardiovascular risk.

**Purpose:** To investigate the cardiovascular burden and vascular inflammation in metastatic breast cancer patients receiving CDK 4/6 inhibitors or everolimus in addition to standard hormonal treatment.

**Methods:** 22 consecutive female patients with metastatic breast cancer were enrolled. Relative wall thickness (RWT) and left ventricle mass (LVM) measurements by transthoracic echocardiography were obtained followed by 24-h ambulatory blood pressure monitoring, and ^18^F-fluorodeoxyglucose positron-emission tomography/computed tomography imaging. Uptake of the radiotracer in the aortic wall was estimated as tissue-to-background ratio (TBR). Each patient was assessed for the aforementioned parameters before the initiation and after 6 months of treatment.

**Results:** At follow up, patients assigned to CDK 4/6 treatment demonstrated increased 24-h systolic blood pressure (SBP) (*p* = 0.004), daytime SBP (*p* = 0.004) and night time SBP (*p* = 0.012) (Group effect). The 24-h mean arterial pressure measurements were also higher in CDK 4/6 population, in comparison to everolimus that displayed firm values (Group effect- *p* = 0.035, Interaction effect-*p* = 0.023). Additionally, 24 h diastolic blood pressure recordings in CDK 4/6 therapy were higher opposed to everolimus that remained consistent (Interaction effect- *p* = 0.010). In CDK 4/6 group, TBR aorta also increased significantly, whereas TBR values in everolimus remained stable (Interaction effect-*p* = 0.049). Both therapeutic regimens displayed statistically significant damaging effect to RWT and LVM.

**Conclusion:** CDK 4/6 inhibitors and hormonal treatment can lead to increased vascular inflammation, and higher blood pressure compared to the combination of everolimus and hormonal treatment. Moreover, both treatment strategies promoted left ventricle remodeling.

## Introduction

Breast cancer has been established as the commonest diagnosed type of cancer in women and a prominent cause of mortality among cancer patients, globally (1, 2). Until early 2020, more than 3.5 million women had a recorded history of breast cancer in the U.S. while almost 300,000 new cases were estimated to be newly diagnosed during the following months (3). Nearly 60% of female patients with breast cancer aged under 50 are hormone receptor (HR)- positive and human epidermal growth factor receptor 2(HER)-negative (4), while the contemporary standard of care treatment in pre- and post-menopausal patients comprises endocrine therapy with the addition of everolimus or cyclin dependent kinases (CDK) 4 and 6 inhibitors (5–7).

Even though hormonal treatment has been studied extensively through the past decades, the crucial role of CDK4/6 pathway inhibition in moderating breast cancer cells propagation and the progress of the disease began to arise in 2015 (8). Many randomized multicentered studies since (PALOMA1-2, MONARCH 3, MONALEESA 2-7) have proven the efficacy and the clinical improvement stemming from this type of targeted treatment (1, 8, 9). Today 3 types of CDK 4/6 inhibitors (palpociclib, ribociclib, abemaciclib) have been approved by the European Medical Association and the Food Drug Administration (FDA) while known adverse effects of this therapy include neutropenia, liver dysfunction, diarrhea (abemaciclib), QTc prolongation (ribociclib), and venous thromboembolism (1, 8–11).

Everolimus is an inhibitor of the mammalian target of rapamycin (mTOR) and specifically of the mTorc1 complex. The mTOR axis is essential in cell multiplication, differentiation, and angiogenesis in breast cancer. Its widespread use in clinical practice emerged after BOLERO 2-3 clinical trials established its efficacy in combination with an aromatase inhibitor in postmenopausal metastatic breast cancer patients (12, 13). Common side effects of treatment are myelosuppresion, non-infectious pneumonitis, hyperglycemia, hyperlipidemia, and hypertension (12, 14).

Both types of therapy might also impair cardiovascular health by means of endothelial injury and vascular dysfunction/inflammation (1, 10, 12, 14–19). Considering the significant overlap between immune and inflammatory response in cancer patients, positron emission tomography (PET)/computed tomography (CT) can be a valuable tool for assessing the post- treatment status of the vasculature, because of its high sensitivity for inflammation detection (20–22). Abnormal values of novel and traditional inflammatory markers such as hsCRP, IL-6, TNF-a, galectin-3, myeloperoxidase (MPO), ST-2, growth differentiation factor (GDF)-15, and microRNAs have been described to be detected in cancer patients with drug induced cardiotoxicity; however it still remains unclear in many cases, whether the inflammatory activation pathway is the result of an ongoing malignancy or a direct result of cardiotoxicity after treatment schemes (23). Moreover, specific widely used biomarkers associated with vascular inflammation in patients receiving CDK 4/6 treatments have not been currently identified and the inflammatory clinical response, with regards to inflammatory assays, is yet to be determined (24). The aim of the current study was therefore, to investigate and compare the cardiovascular and inflammatory impact of CDK 4/6 or everolimus alongside with hormonal treatment in female patients with metastatic HR-positive HER2-negative metastatic breast cancer.

## Methods

This single center prospective observational study included 22 consecutive female patients with metastatic breast cancer that expressed estrogen and/or progesterone receptor and were HER2-negative in a 12 month period. The study protocol was approved by the Alexandra General Hospital review board and ethics committee and each patient provided written consent before the enrollment. Patients with active infection, chronic autoimmune disease, and history of chemotherapy for the metastatic disease and/or adjuvant chemotherapy during the past 3 years were excluded (Total number of patients assessed for eligibility *n* = 31, patients meeting exclusion criteria *n* = 9). All subjects received hormonal treatment and of those, 10 received everolimus and 12 received therapy with CDK 4/6 inhibitors (palpociclib, ribociclib). All patients were free of major cardiovascular events for the past 6 months. Evaluation of left ventricle remodeling, 24 h arterial blood pressure and the inflammation of the aortic wall were performed at baseline and before the initiation of treatment and 6 months after ongoing therapy for both groups. Patients demonstrating increased arterial blood pressure values at baseline measurements were treated according to 2017 ESC/ESH guidelines on arterial hypertension. Hypertensive patients already under treatment before the initiation of chemotherapy continued their standard medication throughout the study protocol.

Complete transthoracic echocardiography (TTE) study was performed using a GE Vivid E9 ultrasound system. The estimation of left ventricle geometry and mass was conducted by 2 experienced operators after careful examination of the acquired images. Relative wall thickness (RWT) was calculated by using the formula: RWT = 2*posterior wall diameter (PWd)/left ventricle end diastolic diameter (LVEDD) while left ventricle mass (LVM) was evaluated using Cube's formula as LVM = 0.8{1.04[([LVEDD + IVSd +PWd]^3^ – LVEDD^3^)]} + 0.6g (IVSd = interventricular septum diameter).

Twenty four hour arterial blood pressure monitoring (24ABPM) was conducted during a usual working day and each patient was advised to act and work normally. Spacelabs 90217 ambulatory blood pressure monitoring (Spacelabs Inc, Redmond, Wash) system was used with a previously described standard protocol (25).

### FDG PET/Ct Imaging

All participants underwent FDG-PET/CT imaging after fasting for at least 12 h prior to the study. None of the patients had blood glucose levels >180 mg dL-1 before injection. FDG was injected intravenously (5MBq/Kg) and scanning was performed at 120 min post injection for vascular tracer uptake assessment. Patients were encouraged to void before imaging and images of the thorax and abdomen were obtained by a hybrid PET/CT scanner (Biograph 6; Siemens, Forchheim). A low dose computed tomography (CT) scan in supine position was obtained, with patients' arms placed above their heads when possible. No CT IV contrast was administered. CT images were acquired with 30 mA, 130 KV, axial slice thickness of 5 mm and table feed rotation of 27 mm per tube rotation. CT radiation exposure was estimated in the region of 5 mSV. PET scanning followed immediately over the same pre-defined body region and the images were reconstructed with a standard Iterative Ordered-Subset Expectation Maximization (OSEM) algorithm using 4 Iterations and 8 subsets. FDG-PET radiation exposure was in the region of 7 mSV for an injected activity of 10 mCi (370 MBq).

### Aortic FDG Uptake Assessment

Aortic FDG uptake was assessed by using previously described validated and reproducible methodology without knowledge of patients' data or laboratory values (26). In brief, regions of interest (ROI) around the aortic wall were manually drawn along the entire aorta in consecutive axial slices at intervals of 5 mm. Metabolic activity within each arterial ROI was measured by maximum standardized uptake value (SUVmax). In the next step, 6 consecutive circular ROIs of 3 mm diameter, were drawn within the superior vena cava and an average venous SUVmean value was calculated. The arterial target-to-background ratio (TBR) was then derived by dividing the mean aortic SUVmax to the average value of venous SUVmean. Finally, aortic TBR was calculated as the sum of TBRs of ascending and descending aorta, aortic arch, suprarenal, and infrarenal abdominal aorta divided by 5.

### Statistical Analysis

Data are expressed as mean ± 1 standard deviation (S.D.) for continuous variables and as percentages for categorical data. The Kolmogorov-Smirnov test was used in order to assess the normality of distributions. Comparisons of baseline variables between groups of treatment were performed utilizing Student's unpaired *t*-test and the non-parametric Mann-Whitney U test as appropriate. Comparisons of continuous paired variables (pre-treatment, post-treatment) were performed utilizing paired *t*-test and Wilcoxon Signed Ranks test as appropriate. To test for changes within and differences between treatment groups after 6 months of treatment, repeated measurement analysis of variance (RMANOVA) was performed with the changes in parameters of ambulatory BP monitoring, echocardiography and PET-scan as dependent variables, and time, treatment group and baseline measurements as fixed parameters. The statistical tests were two-tailed and performed at the 5% level of significance. All statistical analysis was performed using SPSS (Version 20.0, SPSS Inc., Chicago, IL).

## Results

In a cohort of 22 consecutive female patients with metastatic breast cancer, 10 received hormonal therapy with everolimus, and 12 received hormonal therapy with CDK 4/6 inhibitors. Baseline characteristics did not differ significantly between two groups, including blood pressure, body mass index (BMI), and TBR (Table 1). Intra-correlation coefficients (ICCs) with 95% confidence intervals were calculated to test the intraobserver variability (2-way random effects model with absolute agreement), and also to assess interobserver agreement (2-way mixed effects model with absolute agreement) (27) for TBR assessment. The average measure intra-class correlation coefficient (ICC) was 0.996 with a 95% confidence interval from 0.990 to 0.998, *p* < 0.001. The interrater agreement was strong with a 95% confidence interval from 0.884 to 0.992, *p* < 0.001. Concordance correlation coefficients (CCCs) with 95% confidence intervals were calculated to test the intraobserver variability (2-way random effects model with absolute agreement), and also to assess interobserver agreement (2-way mixed effects model with absolute agreement) for TTE measurements regarding LVM and RWT assessment. The average measure intra-class correlation coefficient (CCC) was 0.998 with a 95% confidence interval from 0.996 to 0.999, *p* < 0.001.

**Table 1:** Baseline characteristics.

	**Everolimus (*n* = 10)**	**CDK 4/6 (*n* = 12)**	***p***
Age (years)	62.8 ± 13.6	62.1 ± 17.2	0.925
Hypertension (*n*) ACEi/ARB CCB b-blocker diuretic	4 2 1 3 2	5 3 2 1 2	0.801 0.594 0.571 0.223 0.632
Diabetes (*n*)	1 (IDDM)	1 (NIDDM)	0.943
Dyslipidemia (*n*) Statin use (*n*)	3 2	1 1	0.223 0.429
Smoking (*n*) Previous cancer treatment (*n*) Previous radiotherapy (*n*)	4 (active) 6 2	1 (active) 10 3	0.097 0.221 0.594
24-h SBP (mmHg)	120.6 ± 10.9	130.0 ± 11.2	0.067
24-h DBP (mmHg)	73.9 ± 9.9	73.3 ± 8.3	0.895
24-h MBP (mmHg)	90.3 ± 9.0	93.3 ± 7.3	0.424
Daytime SBP (mmHg)	122.7 ± 11.4	132.4 ± 11.4	0.068
Daytime DBP (mmHg)	75.4 ± 10.6	74.9 ± 8.8	0.910
Daytime MBP (mmHg)	92.3 ± 9.5	95.8 ± 7.0	0.444
Nighttime SBP (mmHg)	113.4 ± 13.5	122.2 ± 14.0	0.160
Nighttime DBP (mmHg)	67.6 ± 10.6	68.3 ± 9.4	0.880
Nighttime MBP (mmHg)	83.3 ± 10.7	87.8 ± 10.8	0.347
TBR aorta	1.87 ± 0.25	1.92 ± 0.32	0.632
EF (%)	54 (8)	55 (5)	0.251
RWT	0.37 ± 0.06	0.39 ± 0.06	0.470
LVM (g)	124.6 ± 36.3	116.9 ± 22.4	0.572

*ACEi, Angiotensin-converting enzyme inhibitor; ARB, Angiotensin receptor blocker; CCB, calcium channel blocker; IDDM, insulin-dependent diabetes mellitus; NIDDM, non-insulin-dependent diabetes mellitus; SBP, systolic blood pressure; DBP, diastolic blood pressure; MBP, mean blood pressure; TBR, tissue-to-background ratio; EF, ejection fraction; RWT, relative wall thickness; LVM, left ventricular mass*.

**Figure 1:**
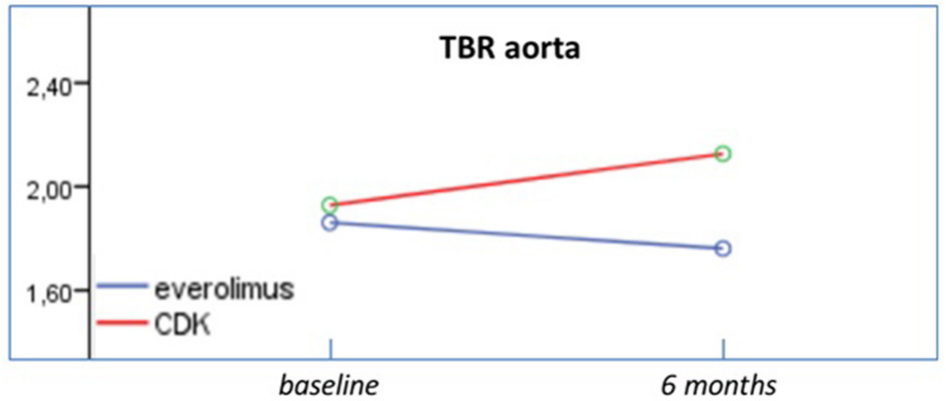
Effects of everolimus and CDK 4/6 on TBR. TBR measurements increased significantly in CDK 4/6 group whereas TBR values in everolimus remained stable at follow up.

**Figure 2:**
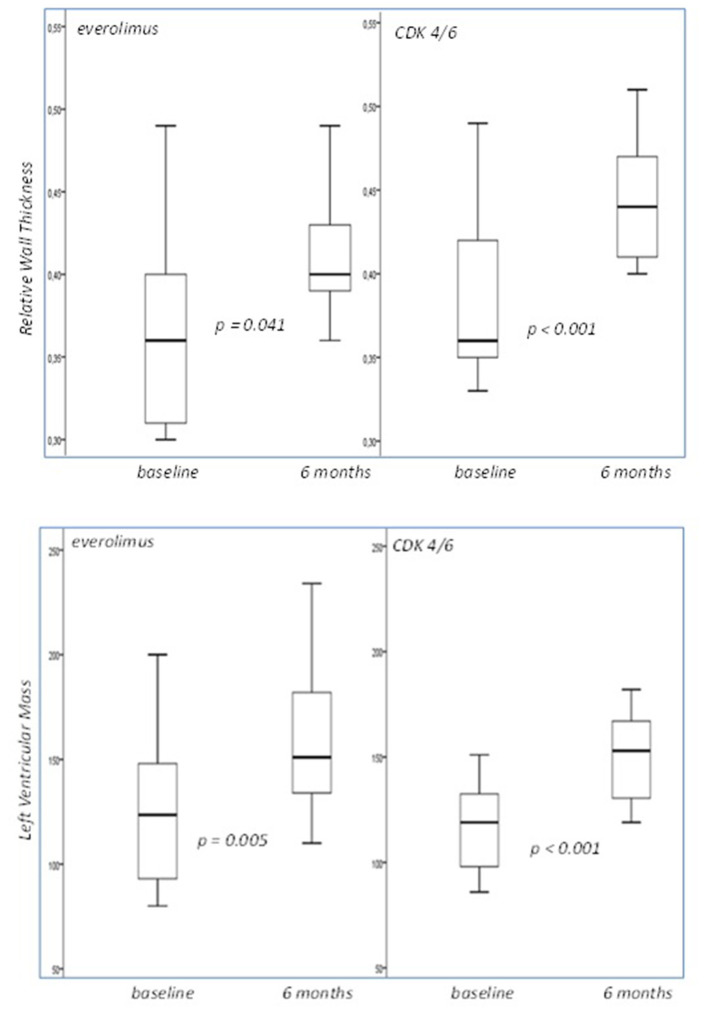
Box plots of the relative wall thickness and left ventricular mass cross-tabulated by different treatment regimens. Both displayed statistically significant damaging effect. LVM was measured in grams.

At follow up, patients assigned to CDK 4/6 treatment demonstrated increased measurements of 24-h SBP (*p* = 0.004), daytime SBP (*p* = 0.004), and night time SBP (*p* = 0.012) (Group effect). The 24-h MAP measurements were also higher in CDK 4/6 population, in comparison to everolimus that displayed firm values (Group effect: *p* = 0.035, Interaction effect: *p* = 0.023). Additionally, 24-h DBP recordings in CDK 4/6 therapy were higher opposed to everolimus that remained consistent (Interaction effect: *p* = 0.010). Profile plots from ambulatory BP monitoring are presented in **Figure 2**. Regarding FDG uptake in the aorta, TBR measurements increased significantly in CDK 4/6 group whereas TBR values in everolimus remained stable at follow up as presented in Figure 1 (Interaction effect: *p* = 0.049). Results of repeated measurement analysis of variance are presented in Table 2.

**Table 2:** Results of repeated measurement analysis of variance for ambulatory blood pressure monitoring and 18-FDG uptake.

	**Everolimus**		**CDK 4/6**		***p***	
	**Baseline**	**6 Months**	**Baseline**	**6 Months**	**Group effect**	**Interaction effect**
**AMBULATORY BLOOD PRESSURE MONITORING**						
24-h SBP	120.6 ± 10.9	119.8 ± 15.9	130.0 ± 11.2	137.9 ± 7.6	0.004	0.139
24-h DBP	73.9 ± 9.9	121.4 ± 15.7	73.3 ± 8.3	141.2 ± 9.7	0.004	0.092
24-h MBP	90.3 ± 9.0	111.2 ± 16.5	93.3 ± 7.3	128.3 ± 9.1	0.012	0.261
Daytime SBP	122.7 ± 11.4	70.7 ± 10.5	132.4 ± 11.4	81.5 ± 8.5	0.161	0.011
Daytime DBP	75.4 ± 10.6	72.2 ± 10.9	74.9 ± 8.8	83.0 ± 10.1	0.185	0.024
Daytime MBP	92.3 ± 9.5	64.3 ± 9.9	95.8 ± 7.0	73.0 ± 8.3	0.215	0.063
Nighttime SBP	113.4 ± 13.5	88.9 ± 12.9	122.2 ± 14.0	102.2 ± 8.0	0.036	0.023
Nighttime DBP	67.6 ± 10.6	90.5 ± 13.5	68.3 ± 9.4	104.0 ± 9.7	0.042	0.035
Nighttime MBP	83.3 ± 10.7	81.9 ± 12.7	87.8 ± 10.8	93.4 ± 7.4	0.054	0.166
**18-FDG PET**						
TBR	1.86 ± 0.28	1.76 ± 0.16	1.92 ± 0.32	2.13 ± 0.36	0.089	0.049

*DBP, diastolic blood pressure; MBP, mean blood pressure; SBP, systolic blood pressure mmHg; TBR, tissue-to-background ratio*.

Both therapeutic regimens displayed statistically significant damaging effect with regards to the following echocardiographic variables: RWT and LVM (Figure 2). On the contrary, ejection fraction did not significantly change in both groups (from 54 to 50% for Everolimus, *p* = 0.109, and from 55 to 55% for CDK 4/6 group *p* = 1.000).

## Discussion

CDK 4/6 treatment strategy promoted vascular inflammation by means of increased TBR values, higher blood pressure values as recorded by 24 h ABPM and induced left ventricle remodeling. CDK4/6 inhibitors function as ATP competitive inhibitors, while they intervene in the phosphorylation and inactivation of retinoblastoma, a key tumor suppressor fundamental in cell cycle and the inactivation of FOXM-1. Thus, moderation of breast cancer cells proliferation occurs, without directly causing their apoptosis, and enhance their capacity to present antigen and stimulate cytotoxic T cells (1, 9, 15).

CDK inhibition has been found to trigger interferon production and indirectly moderate cytotoxic T cells activation against tumor cells proliferation (15). CDKs are widely expressed in breast cancer cells and play a crucial role in the initiation of an inflammatory cascade comprising IL-8, IL-6, VEGF-A, and others (28). However, specific blockade of certain kinases (4 and 6) in the context of metastatic breast cancer might lead to compensatory upregulation or do not alter at all the function of other members of CDK family such as 7 and 9, kinases that have been proven to regulate neutrophil apoptosis and promote inflammatory response (17). Additionally, novel findings of new interstitial lung disease, pneumonitis, and inflammation in patients receiving CDK 4/6 inhibitors support the notion that the inflammatory pathway is not blocked adequately with regards to this group of patients and moreover can exacerbate inflammatory response (16). The above cumulative reports led recently the FDA to issue an official safety announcement warning about the complete class of CDKI (29).

Hypertensive response as a result of an inflammatory process has been described extensively (18, 19, 30). Chronic inflammatory activation has been implicated to the dysregulation of angiotensin II axis, sodium retention, and increased sympathetic outflow. The stimulation of angiotensin-aldosterone pathway and catecholamines promote reactive oxygen species (ROS) production in vasculature thus enhancing chemokine and adhesion molecules fabrication. Moreover, activated T cells interact with macrophages and leukocytes resulting in the activation of other inflammatory assays, such as IL-6, TGF-β, and the production of IL-17 and other cytokines by direct T cells. The above changes promote further ROS production, sodium retention and vasoconstriction (30–33).

The increased RWT and LVM values recorded in the group of CDK4/6 inhibitors can be interpreted as a result of ventricle remodeling and in the context of an inflammatory induced hypertensive state. Many studies have proposed the role of cytokines such as TNF-a, IL-1, and IL-6 in alterations of left ventricle geometry and progressive diastolic and systolic functional impairment (34–36). Concurrently, increased ventricular wall stress as a result of a raised systemic afterload (in cases such as hypertension) has been found to promote further release of inflammatory cytokines in systemic circulation leading to further remodeling and geometry alterations that might lead to severe diastolic and systolic dysfunction (cytokines pleiotropic effect-positive feedback mechanism) (36, 37).

The group that received Everolimus did not demonstrate significant alterations at follow up in TBR and 24 h ABPM recordings. Everolimus interacts exclusively with the mTORC1 compound (direct inhibition) and promotes phosporylation of P70 ribosomal S6 protein kinase. Furthermore, it blocks HIF-1 expression and moderates angiogenesis with an impact on VEGF and smooth muscle and endothelial cells propagation. As an anticancer agent it does not promote direct cardiotoxicity; its impact on vasculature stems from hyperglycemia, hyperlipidemia and hypertension that it might induce (12–14).

In the present study however, RWT and LVM were increased at 6 months follow up. Despite the fact that established hypertension was not apparent in this group, the above findings support the theory of concentric remodeling of the left ventricle in the context of possible microvascular dysfunction induced by Everolimus treatment (14) and an ongoing chronic inflammatory process(metastatic breast cancer) promoted by the cytokine pathway (34–36).

### Study Limitations

The present study exhibits the results of a single center observational report including a relatively limited number of patients. Secondly the study protocol did not include the measurement of inflammatory markers.

## Conclusion

The findings of the present study suggest that both treatment strategies might impair cardiovascular function. Specifically, CDK 4/6 inhibitors and hormonal treatment promotes vascular inflammation, hypertensive response, and alters left ventricle geometry. On the contrary, Everolimus and hormonal treatment does not have such a compounding impact on cardiovascular burden, by means of TBR and 24 h ABPM measurements, although left ventricle concentric remodeling was noted in TTE at 6 months of follow up.

To our knowledge, this is the first study to assess the cardiovascular impact of contemporary anti-neoplasmatic treatment in metastatic female breast cancer patients with HR-positive HER2-negative phenotype, using a combination of different techniques including PET-CT imaging. Taking into consideration the above findings, the authors have the notion that close monitoring with TTE and ABPM at baseline and during treatment would be a reasonable approach in this subgroup of breast cancer patients. Moreover, long term consequences of increased vascular inflammation might be accessed further with the implementation of peripheral vascular imaging and/or aortic and peripheral arteries functional alterations, assessed by well-established methods such as IMT (intima media thickness), ABI (ankle branchial index), and/or PWV (pulse wave velocity), respectively, especially in cases with emerging signs and symptoms of cardiovascular dysregulation. Both cardiologists and oncologists, ought to be alert and in close collaboration for the prompt detection of cardiovascular toxicity when treating breast cancer patients receiving either CDK 4/6 inhibitors or Everolimus.

## Data Availability Statement

The raw data supporting the conclusions of this article will be made available by the authors, without undue reservation.

## Ethics Statement

The studies involving human participants were reviewed and approved by Alexandra General Hospital Review board and Ethics committee, Athens, Greece. The patients/participants provided their written informed consent to participate in this study.

## Author Contributions

All authors listed have made a substantial, direct and intellectual contribution to the work, and approved it for publication.

## Conflict of Interest

FZ reports honoraria for lectures and has served in an advisory role for Astra-Zeneca, Daiichi, Eli-Lilly, Merck, Novartis, Pfizer and Roche. MD reports consulting fees, lecture fees, and honoraria from Janssen, Amgen, Celgene, and Takeda; research funding from Janssen, Amgen, Takeda, and Genesis Pharma; and consulting fees from Bristol-Myers Squibb. The remaining authors declare that the research was conducted in the absence of any commercial or financial relationships that could be construed as a potential conflict of interest.

## Supplementary Material

Supplementary Figure 1 Indicative comparative images of ^18^FDG PET/CT scanning between the two groups.
