# Supplementary figures and images for: Vascular Inflammation and Cardiovascular Burden in Metastatic Breast Cancer Female Patients Receiving Hormonal Treatment and CDK 4/6 Inhibitors or Everolimus
Source: Front Cardiovasc Med. 2021 Feb 23;8:638895. doi: 10.3389/fcvm.2021.638895 (PMC7959765; doi:10.3389/fcvm.2021.638895)

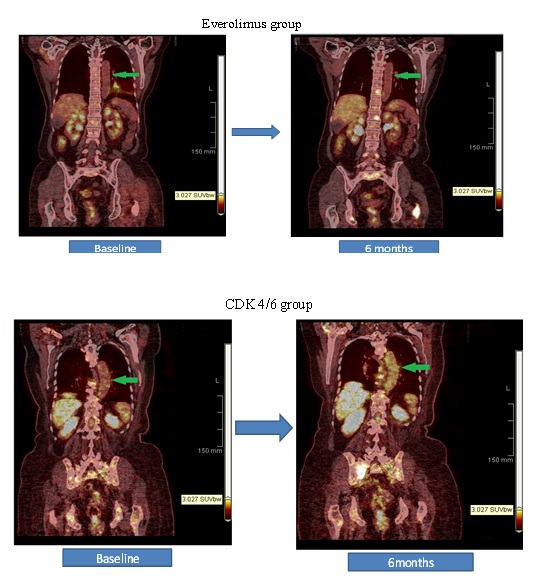

Supplement: Supplementary Figure 1 — Indicative comparative images of 18FDG PET/CT scanning between the two groups. [file Image_1.JPEG]
